# In data we trust: X-ray diffraction experiments for charge density investigations

**DOI:** 10.1107/S205252062300776X

**Published:** 2023-09-19

**Authors:** Regine Herbst-Irmer

**Affiliations:** a Institut für Anorganische Chemie, Georg-August-Universität Göttingen, Tammannstraße 4, Göttingen, Lower Saxony 37077, Germany

**Keywords:** data quality, charge density, multipole model, topological analysis of the electron density

## Abstract

A short commentary is given on the paper by Vosegaard *et al.* [ *Acta Cryst*. (2023), **79**, 380–391], which compares charge density models derived from four datasets measured on conventional diffractometers with the results of a high-quality dataset from SPring-8.

Investigations into charge density focus on deviations from spherical valence density to describe the bonding situation in a specific structure. The Hansen & Coppens (1978 ▸) multipole model can be used to describe this aspherity, because this model partitions the electron density in the core, the spherical valence density and the aspherical valence density. This model needs more parameters compared to the independent atom model which uses just spherical atomic form factors. The deviations from spherity are very small compared to the electron density from the core. Therefore, excellent high-resolution data are necessary. The information about the valence density is mainly contained in the low-order data, while high-resolution data are needed to deconvolute aspherical atomic electron density from thermal vibrations. The data need to be of very good quality along the whole resolution range, and especially the low-order data need to be collected with high accuracy.

In recent decades, there have been numerous new developments in the fields of sources and detectors. In terms of detectors, technology has progressed from point detectors to CCD and nowadays energy-discriminating pixel detectors, which greatly reduced the data collection time and which foster spectral purity. The development in sources from sealed tubes to microsources, rotating anodes, MetalJet to synchrotrons radiation supplies more and more beam intensity. There is a strong tradition to examine which of these new developments really provide better data for charge density investigations, *e.g.* Martin & Pinkerton (1998 ▸), Coppens *et al.* (2005 ▸), Jørgensen *et al.* (2014 ▸), Wolf *et al.* (2015 ▸), Graw *et al.* (2023 ▸), Ruth *et al.* (2023 ▸). Higher resolution is not enough, excellent low-order data are also needed, and the question is which combination of hardware leads to reliable statements about the bonding situation in a particular compound.

This tradition continues with the paper of Vosegaard *et al.* (2023 ▸). The authors compare data collected at 100 K from new in-house diffractometers, a Rigaku Synergy-S (Mo and Ag source, HyPix100 detector) and a Stoe Stadivari (Mo source, EIGER2 1M CdTe detector) and an older Oxford Diffraction Supernova (Mo source, Atlas CCD detector) to an investigation with excellent synchrotron data collected at 25 K and λ = 0.248 Å at the BL02B1 beamline at SPring-8 (Vosegaard *et al.*, 2022 ▸). As the test structure, melamine was selected.

First, the authors investigate the data quality by comparing figures of merit such as *R*_int_ and *I*/σ. Of course, the SPring-8 data win concerning the highest possible resolution and lowest collection time. However, considering the variation of *R*_int_ and *I*/σ over the whole resolution range, the modern diffractometer Synergy-S and Stadivari can keep up to *circa* 0.9 Å^−1^, while the older Supernova delivers noisier data. This is probably due to the virtually noise-free pixel detectors compared to the older CCD technology. However, all data, even from the Supernova, could be used to a resolution up to at least 1.17 Å^−1^.

The derived unit-cell parameters of the four datasets at 100 K differ only a little – with a small feature that the differences between the values derived from the Synergy Mo and Ag as well as those from the Supernova and the Stadivari are smaller than the differences between these two groups.

For the refinements (Volkov *et al.*, 2006 ▸), an *I* < 3σ intensity cutoff was used, leading to a very different amount of used data in the five datasets. Although the data to parameter ratio is larger than 10 in all refinements, I wonder if, especially for the Supernova dataset, overfitting could be excluded (Krause *et al.*, 2017 ▸), which the authors had tested for the SPring-8 data but not for the new datasets. All five datasets deliver low *R* factors (≤ 3.4% for Supernova and < 1.7% for the other datasets) and min/max residuals (≤ ±0.25 e Å^−3^ for Supernova and < ±0.15 for the other datasets), *DRK* plots with the value 1±5% across the whole resolution range (Zavodnik *et al.*, 1999 ▸; Zhurov *et al.*, 2008 ▸), and Henn–Meindl plots (Meindl & Henn, 2008 ▸) with virtually no features.

*U*_eq_ values for the heavy atoms agree well, while the values for hydrogen atoms derived from Hirshfeld atom refinement (Capelli *et al.*, 2014 ▸; Fugel *et al.*, 2018 ▸; Kleemiss *et al.*, 2021 ▸) from the Supernova data deviate significantly from those of the other three datasets.

The derived multipole parameters show the same trend for all five datasets, although the differences for the carbon atom parameters are significant. The derived monopole population even spread between 4 and 4.5 e, indicating slightly negatively charged carbon atoms. However, the Bader charges are more sensible with slightly positively charged carbon with a small spread of 0.2 e and negatively charged nitro­gen atoms. In addition, the topological values ρ_BCP_ and ∇^2^ρ_BCP_ are in good agreement except for the Supernova data, where the bond critical point of one weak π–π interaction could not be found.

Overall, it was found that even with conventional diffractometers and good quality crystals of small organic molecules reliable electron densities can be achieved with the multipole model. However, the better the data the more features can be seen: only for the excellent SPring-8 data set hydrogen κ values could be refined, while weak interactions could not be found with the weakest Supernova data.
